# Post COVID-19 lockdown: measures and practices for dental institutes

**DOI:** 10.1186/s12903-020-01281-6

**Published:** 2020-10-27

**Authors:** Sausan Al Kawas, Natheer Al-Rawi, Wael Talaat, Zaid Hamdoon, Basheer Salman, Saad Al Bayatti, Waseem Jerjes, A. B. Rani Samsudin

**Affiliations:** 1 Department of Oral and Craniofacial Health Sciences, College of Dental Medicine and University Dental Hospital Sharjah, University of Sharjah, P.O. Box 27272, Sharjah, UAE; 2 Research Institute of Medical and Health Sciences, University of Sharjah, Sharjah, 27272 United Arab Emirates; 3 Department of Oral and Maxillofacial Surgery, Faculty of Dentistry, Suez Canal University, Ismaillia, 41522 Egypt; 4 North End Medical Centre, Hammersmith and Fulham Partnership, National Health Service, London, W14 9PR UK

**Keywords:** Dental institutes, Post COVID-19, Protective measures, Risk of COVID-19 transmission, Dental practice, Healthcare workers

## Abstract

Resuming regular clinical activities at dental premises after the COVID-19 lockdown period or post COVID-19 is likely to be a challenge for all dental institutes. When returning to the dental practice or training, staff and students alike should abide by the new rules and regulations. In the process of controlling viral spread, clinical dental facilities face a higher risk of disease transmission among patients as well as clinical and non-clinical staff. Aerosols formation and diffusion into the surrounding air can be a real concern of viral transmission, if no protective measures are established. We aim in this review to present the currently implemented measures and propose changes in clinical dental facilities to minimize the risk of transmission. Dental professionals should be prepared to treat every patient as a suspected COVID-19 carrier and be ready to receive and manage an overwhelming number of patients. We suggest that dental practices establish a sensible workforce shift schedule, improve ventilation levels, reduce dental aerosol generating procedures, and develop a comprehensive guidance to Healthcare Workers to reduce the risk of COVID-19 transmission.

## Background

The World Health Organization (WHO) declared the novel Coronavirus disease 2019 as a Public Health Emergency of International Concern on the 30th of January 2020 [1]. On the 11th of March 2020, the WHO announced that the coronavirus outbreak was a pandemic. The number of cases progressively increased and easily spread over the world via aerosols produced by an infected person or close contact. By the end of September 2020, COVID-19 was confirmed to affect almost all the world countries and more than 33 million individuals worldwide and cost us about one million lives, as reported by WHO [2]. Although the total number of cases and death are on the rise worldwide, almost all countries have removed their lockdown measures with instructions on keeping social distaining and maintaining all Infection Control procedures [3].

There are real concerns of a second wave following the lifting of lockdown measures in many countries [4]. The epidemiologic data is being continuously updated as the epidemic evolves around the world. During previous severe acute respiratory syndrome coronavirus (SARS-COV1) outbreaks, human to human transmission occurred through droplets, contact and fomites, similar to COVID-19. It is believed that most of the COVID-19 infected individuals will be either asymptomatic or suffer from mild symptoms, with some suffering from acute cardiac events, severe lower respiratory tract infections, acute respiratory distress syndrome, renal failure, and other pro-thrombotic events which ultimately can lead to death in certain group of people [5]. Healthcare workers are at a very high risk of acquiring infections by the new SARS COV-2 during provision of care for patients who carry the virus. Many healthcare professionals around the world are succumbing to the complications of COVID-19 despite many being young and having no underlying health problems. This is thought to be related to the increase in the viral load which triggers the immune system into cytokine storm that can lead to wide spread damage resulting in multi-organ failure [6].

Studies have shown that respiratory viruses can be transmitted from “person to person” through direct or indirect contact, or through coarse or small droplets, including saliva, and it is believed that COVID-19 is no exception to this rule [7]. It is well documented that virus transmission can occur through aerosols generated during medical and dental procedures [8, 9]. Hence, this review presents the currently implemented measures and propose changes in clinical dental facilities to minimize the risks of COVID-19 transmission in the “new normal” clinical dentistry.

## Measures on administrative control

Resuming regular clinical practice at dental premises after the lockdown period post COVID-19 is likely to be a challenge from an administrative perspective. It is important to work on the assumption that the pandemic will not fully resolve and the possibility of a second wave. In fact, returning to pre COVID-19 era will likely take years unless an effective and safe vaccine is developed and disseminated worldwide. When resuming clinical practice of dentistry post COVID-19, it is expected that the influx of patients will increase. The challenge here is to regulate the flow of the patients and prioritize care to ensure social distancing measures are followed. Simultaneously, it is important to reduce patient’s suffering and potential complications from long waiting times. When returning to the “new normal” dental practice and training at colleges and/or institutes, all staff and students would be expected to abide by the new rules and regulations. This includes adequate social distancing, reduced gatherings in buildings, in clinical and educational settings.

### Procurement

Preparing for the “new normal” should involve the procurement of specific equipment, which is not usually available in a dental clinic setting such as pulse oximeter, N95 mask, and face shields. Acquiring this equipment from the current market may represent a challenge due to the high demand, hence the sooner the process is initiated, the higher is the probability of success. One example here is the procurement of facial masks. Providing N-95, FFP2 or FFP3 masks is essential, as there is controversial information on the efficacy of the regular surgical face masks in preventing the transmission of disease to the individual who wears it [10]. Good hand washing (for 30 s) and the use of surface disinfectants have been recommended as the most effective measure in reducing the risk of transmission at any setting, thus, the timely procurement of such consumables is critical [11]. Another example is the need to acquire infrared thermometers to screen patients at the reception area. The availability of WHO-approved COVID-19 testing kits to be used with suspected cases will be advantageous in any practice. The well planned procurement of such new essentials is extremely important for the “new normal”.

### Establish appropriate workforce schedule

It is expected that the return to the new normal will lead to a spike in the number of patients seeking dental care in the first few weeks if not months. Hence, it is extremely important to establish an “adaptive” clinic schedules that will ensure that social distancing measures are respected. For example, having one hour of overlap between shifts provides flexibility and facilitates the handover, which reduces the workload stress and the possibility of viral spread [12]. It is important to pay attention to the patients coming for dental care and categorize them according to their risk of developing complications if they are infected by COVID-19.

When it comes to clinical staff that are considered in the “at high risk” category, it is advisable that they receive a modification to their daily roles to ensure that they have no contact with anyone who can put them at risk of contracting the virus. While, clinical staff that are considered in the “at risk category” need to practice strict social distancing during this pandemic. Ideally this needs to be employed until an effective vaccine is trialed and approved by the respective regulatory bodies [13].

In case any of the clinic staff testing positive for COVID-19, the staff member should self-isolate for a period of 14 days as per the Occupational Safety and Health Administration (OSHA) guidance and contact tracing should be initiated [13]. It is worth remembering that self-isolation period can vary from country to country and the contact tracing can be a real challenge as, in one of the studies, the virus was isolated from the patients’ secretions after a range of 8–37 days [14]. But it remains the consensus that a person is unlikely to be infectious after self-isolating for 14 days since developing any COVID-19-related symptoms [14].

### Patients’ scheduling and triaging

Patients’ scheduling and triaging are critical parameters to be managed before the re-starting of the new normal. Patients can be advised to wait in their vehicle till patient's turn comes, however this could be a challenge where other modes of transportations have been used. It has been advised that keeping people away from each other by two meters can significantly reduce the risk of transmission of COVID-19 [15]; however, some countries have applied 1 m or 1.5 m social distancing rule [16]. An interesting study suggested that aerosols can travel 6 m or more when someone is coughing or sneezing [17]. While another study has shown that the virus can remain viable and infectious in aerosols for hours which renders social distancing measures less effective [18]. Despite all that, it is widely accepted that social distancing measures reduce the infection rate and should be employed to keep everyone safe [19].

In reception and waiting areas, modifications should be made to ensure that there is two-meter distance between patients. Proper ventilation of the waiting area should be ensured and air circulation should be prevented to reduce viral spread. All unnecessary accessories (e.g. gadgets, books and magazines) should be removed from waiting areas and patients should be advised to come alone except for children and elderly. Appointments should be spread throughout the day to ensure less people in the waiting area at any time. Patients should be advised to wear face masks or face shields if they cannot wear a mask. Teledentistry can also be integrated into routine dental practice to minimize the risk of direct face-to-face contact with patient. It is a valuable tool in reducing the risks of disease transmission by facilitating remote dental triage, diagnosis, consultation, and patients’ education via the use of information technology [20].

Scheduling of the patients should be organized so that enough time for decontamination of the clinics is available between cases, especially following any aerosol generating procedures. Extensive environmental contamination was documented in one study of a single COVID-19 patient who had mild respiratory symptoms; however post-decontamination samples were negative, signifying the critical role of clinic decontamination in preventing cross infection [21].

Modified structured telephone and online triaging should be employed as a method to reduce the risk of disease transmission inside the clinic by reducing the number of patients present at any time. Patients should be scheduled according to their risk status. Appointments for high-risk patients should be scheduled at the end of a morning shift or at the end of an evening shift. In addition, an initial assessment by nurses in the assessment room should be established at every dental clinic. At this initial screening, all individuals entering the clinic should complete a COVID-19 risk assessment questionnaire, should have a remote check of their temperature and should be provided with a mask to wear inside the clinic, if not wearing one already.

Clinical waste volumes are expected to increase due to the increase in the use of PPE, and the clinical waste from any suspected patient should be treated as suspected COVID-19 clinical waste. Staff should wear scrubs while at work, and all staff scrubs should be cleaned/decontaminated at a medical laundry on daily basis.

### Contact tracing

Contact tracing is an effective method to contain outbreaks. This intervention played a major role in the past to control previous pandemics (i.e. SARS, Ebola and MERS) [22–24]. Dental electronic patient records should be updated to enable recording information to facilitate contact tracing of suspected or confirmed COVID-19 cases. This measure will assist public health authorities trace, test and quarantine individuals who test positive. The role of dental clinical practices is to record as much patients’ identifying information as possible in their medical records systems, so that this information can be provided to the authorities when needed. This data will be critically valuable in case of an unfortunate second wave event of COVID-19.

## Guidelines on facility engineering control

Clinical engineering controls, such as installing high-efficiency air filters, raising ventilation levels, and providing negative ventilation pressure, can reduce exposure to hazards. The Centers for Disease Control and Prevention (CDC) recommends that patients with known or suspected COVID-19 should be placed in an airborne infection isolation room, if available [25].

The isolation room should be used for patients with or suspected of having an airborne infection that require aerosol generating procedures, and the use of an enclosed hood that is engineered to provide complete isolation is suggested. It is essential to ensure that heating, ventilation, air conditioning (HVAC) filters are properly installed and regularly maintained to prevent air leakages and dust overloads. The air should also flow from halls and corridors (cleaner areas) into the isolation rooms (less clean areas) [26]. This prevents cross-contamination and spread of the virus. Regular daily monitoring of the air pressure periodically with audible or visual control system is mandatory. As the first choice, the exhausted air must be drenched directly outside the building. If this is not feasible, until recirculation, the air should be passed through a filter with high-efficiency particulate air (HEPA) [27]. If the exhaust air is re-circulated, it is suggested to have a high density HEPA filter with a minimum efficiency reporting value (MERV) of 18 or higher and the doors should be kept closed as much as possible [28]. Barriers such as glass or plastic windows may be an important remedy for reducing Health Care Personnel (HCP) exposures to patients that are potentially contaminated. This method may be useful in reception areas where patients can first report to a healthcare facility upon arrival.

## Guidelines on staff education and training

One of the potential challenges today is the training of healthcare workers on the prevention of the transmission of infections in healthcare facilities [29]. Hand hygiene is by far the most important procedure in the Standard Precautions. Dental Health Care Personnel (DHCP) should be able to recognize hand hygiene as a critical component of infection prevention and control, properly perform hand hygiene and glove use during patient care activities. DHCP must always be trained to the correct way to wash hands with soap and water or with an alcohol-based hand rub according to the WHO-recommended method [30]. The CDC recommends using alcohol-based hand sanitizer greater than 60% ethanol or 70% isopropanol in healthcare settings. Unless hands are visibly soiled, an alcohol-based hand rub is preferred over “soap and water” in most clinical situations due to scientific evidence of better compliance [31]. It is important that DHCP instruct the patients, before any procedure, to use antimicrobial mouth rinse for 30 s with a hydrogen peroxide solution 1% or iodopovidone 0.2% [32]. It is also important to make informed clinical decisions and educate the public to prevent panic while promoting the health and well-being of our patients during these challenging times [33].

## Guidelines on clinical dental practice

The emergence of SARS, MERS and currently the COVID-19 pandemic led to development and inclusion of respiratory hygiene/cough hygiene as a component of the Standard Precautions. However, in cases of these viruses’ outbreak, the Standard Precautions during clinical dental practice must be modified in order to eliminate or contain aerosol production during patient care.

### Personal protective equipment (PPE)

While correctly using PPE can help prevent some exposures, it should not replace other prevention strategies. Examples of PPE include: gloves, goggles, face shields, facemasks, and respiratory protection, when appropriate. PPE must be selected based on the scientific evidence supporting its use in that profession and should consistently and properly used during the pandemic. Robust regular inspection, maintenance and replacement should be carried out as necessary. The use of PPE post COVID-19 is unlikely to change for some time or until a vaccine is found [7].

The use of barriers (e.g., gloves, gowns, head caps, masks), sterilizing devices, equipment and clothing, and implementing environmental control (e.g. surface processing protocols and health service waste handling), as well as handling and adequate processing of sharp instruments are mandatory in dealing with suspected or confirmed COVID-19 cases [34]. The currently used personal protective equipment for healthcare workers for providing patient care in dental setting are classified into four levels [7]. Depending on occupational class, age of healthcare worker and their medical co-morbidities and other factors, this would usually provide recommendation on their suitability for work or not, and if any work environment changes need to be carried out (Table 1) [13]. Clinical judgment and decisions should be based on the local and/or regional infection status (pandemic or epidemic infection), suspected/confirmed infectious agent, severity of the illness caused, transmission route of the infectious agent, and the care setting and procedures undertaken. The suggested PPE modifications according to the risk are listed in (Table 2) [35].

**Table 1:** Occupational risk levels during the pandemic. The classification of health workers in dental setting into four levels depending on occupational class, age, their medical co-morbidities, and other factors [13]

Risk levels	Highly critical (Any 3 factors) Level A	Critical Level B	Semi critical Level C	Non critical Level D
Occupational class	Dentists, Dental Students, Nurses	Dentists, Dental Students, Nurses	Non clinical nurses, lab technician	Receptionists, Security personal, waste workers and cleaners
Age	Above 70 s	Less than 50 s	Less than 50 s	Less than 50 s
Medical Status	Immune deficiencies or upper respiratory track disease	Medically fit*	Medically fit	Medically fit
Geographical area	Countries which declared as epicenter of the disease	Any country involved with COVID-19	500–1000 new case/ daily	500–1000 new case/ daily
High-population-density work environments	Hospital	Hospital, or dental clinic	Hospital, or dental clinic	Hospital, or dental clinic
Work modification	Advised not to work	Standard + contact + enhanced airborne precautions	Standard precautions	Standard precautions
Level modification	N/A	N/A	It can be B if: Above 70 s and with Medical problem or living in epicenter of infection	N/A

*In case of immunologically compromised health worker, restricted to non aerosol generating dental procedures

**Table 2:** The necessary PPE modifications according to occupational risk modifications of PPE according to the four levels of occupational risk [35]

PPE modifications According to risk level	Level 1 (Non pandemic or epidemic), non-suspected case	Level 2, A (Pandemic or epidemic) with non AGP treatment	Level 2, B (suspected) with AGP treatment	Level 3 Diagnosed case
Good hand hygiene (Alcohol based)	Yes (Alcohol or Soap)	Yes	Yes	Yes
Disposable gloves	Yes	Yes	Yes	Yes
Non fluid resistance apron	Yes	Yes	No	No
Fluid resistance apron gown	No	No	Yes	Yes
Fluid resistance mask (IIR)	Yes	Yes	No	No
Fluid resistance filtering face piece (FFP3)	No	No	Yes	Yes with powered hood respirator
Eye protection	Consider (if risk of spraying or splashing)	Yes	Yes	Yes
Face shield	No	No	Yes	Yes
Disposable fluid-resistant hood	No	No	Only head cap	Yes
Surgical wellington boots or closed shoes	No	No	No	Yes
Disposable boot covers	No	No	No	Yes
Program involved	Standard precautions	Standard + contact + droplet precautions	Standard + contact + enhanced airborne precautions	Standard + contact + extended airborne precautions

### Aerosol generating procedures (AGP)

It is suggested to reduce dental Aerosol Generating Procedures (AGP), however, if an AGP is a clinical necessity, the use of high-power suction and rubber dam should be implemented where possible. Extraoral vacuum aspirators have been shown to have superior capture compared to intraoral devices. Many studies have effectively demonstrated the rationale behind using extraoral vacuum aspirators as one of several strategies to decrease the amount of airborne pathogens, such as the SARS COV-2 virus [36]. The dental practitioner should aim to complete the treatment in one visit, where possible. Non-AGP treatments should be carried out as usual with strict compliance with the standard Infection Control procedures. This will ensure that there is no contact or droplet transmission of COVID-19. Eye protection, disposable fluid-resistant surgical mask, disposable apron and gloves should be worn while carrying out any procedure [35].

## Conclusions

During the COVID-19 lockdown, the authors have implemented an emergency operations workflow at the University Dental Hospital, abiding to the local directives of the health authority. This workflow involved limiting the admission to cases of severe pain, bleeding and swelling. Following the lockdown, the authors have implemented the same measures presented in this study, which are still in accordance with the directives of local health authorities. Thus, we have presented here an overview and provided practical solutions to be carried out in the post COVID-19 pandemic in a dental setting, at least until identifying an effective vaccine. The dental professionals and staff should be prepared to treat every patient as a suspected COVID-19 carrier and be ready to receive and manage an increased number of patients requiring care when services are resumed. We suggest that dental practices establish a sensible workforce shift schedule and manage patients’ scheduling and triaging. It is proposed that dental clinics should aim to improve ventilation levels by installing high-efficiency air filters and providing negative pressure isolation room. It is also suggested to aim to reduce dental aerosol generating procedures in dental settings as much as possible. The authors acknowledged the different approaches employed by healthcare regulatory organizations due to variations in epidemiological situations and national public health strategies in different countries and geographical locations [32]. The development of a comprehensive guidance to healthcare workers with ongoing updates will reduce the burden on the dental clinics to face the post COVID-19 challenges.

## Data Availability

The datasets used and/or analyzed during the current study are available from the corresponding author on reasonable request.

## Abbreviations

WHO: World Health Organization
OSHA: Occupational Safety and Health Administration
CDC: Centers for Disease Control and Prevention
HVAC: Heating, ventilation, air conditioning
HEPA: High-efficiency particulate air
HCP: Health Care Personnel
DHCP: Dental Health Care Personnel
PPE: Personal Protection Equipment
AGP: Aerosol Generating Procedures
